# ‘I Would Rather Be Having My Leg Cut off Than a Little Needle’: A Supplementary Qualitative Analysis of Dentally Anxious Children’s Experiences of Needle Fear

**DOI:** 10.3390/dj8020050

**Published:** 2020-05-13

**Authors:** Fiona Noble, Jennifer Kettle, Joe Hulin, Annie Morgan, Helen Rodd, Zoe Marshman

**Affiliations:** 1 Charles Clifford Dental Hospital, Sheffield S10 2SZ, UK; 2 School of Clinical Dentistry, University of Sheffield, Sheffield S10 2TA, UK; j.e.kettle@sheffield.ac.uk (J.K.); a.g.morgan@sheffield.ac.uk (A.M.); h.d.rodd@sheffield.ac.uk (H.R.); z.marshman@sheffield.ac.uk (Z.M.); 3 Mental Health Research Unit, School of Health and Related Research, University of Sheffield, Sheffield S1 4DA, UK; j.hulin@sheffield.ac.uk

**Keywords:** needle fear, quality of life, dental anxiety, children, supplementary analysis

## Abstract

Fear of needles is common in childhood, with up to 50% being affected to some degree. In individuals who are dentally anxious, the prevalence may be as high as 91%. Fear of needles, and therefore intra-oral injections can have negative impacts on children’s quality of life and healthcare experiences, including a requirement for pharmacological methods to facilitate dental treatment. The aim of this study is to identify whether dentally anxious children report fear of injections and explore how these children experience a fear of needles in a dental setting. A supplementary analysis of interviews collected as part of two previous studies relating to children with dental anxiety. Five main themes were identified: feelings about needles; the nature of needle fear; the context of the fear, its consequences and how children tried to control the process. Children showed a desire to have control of their healthcare interventions, and wanted to trust the healthcare professionals giving the injections. There is evidence that children with dental anxiety also experience fear of needles, including intra-oral injections. Further primary qualitative research is needed to explore this topic in more depth and to design appropriate child centred interventions to reduce needle fear.

## 1. Introduction

Fear of needles or injections is common, with a recent systematic review by McLenon and Rogers concluding that it affects up to 50% of children and adolescents [1]. In adults, the prevalence varies from 9–30% [1,2,3,4]_._ Needle fear can develop as a result of a traumatic experience, and can also be a learned behaviour, with parents who have a fear of needles often passing this fear onto their children [5]. Throughout the literature this fear is often interchangeably described as needle or injection fear, anxiety or phobia. For the purposes of this paper, the umbrella term ‘needle fear’ will be used to encompass these terms. Needle fear can be a barrier to receipt of healthcare interventions, including routine vaccinations for preventable serious diseases such as hepatitis B and measles, blood tests and intravenous medications [5,6]. Fear of intra-oral injections has been reported as a specific barrier to both adults and children receiving dental treatment [7,8]. The concept of needle fear amongst those with dental anxiety is not a new one, with a 1988 Japanese study identifying the injection as one of the most stressful elements and a 1995 study of low income American families confirming that injection is a main theme in relation to dental anxiety in children [9,10]. McLenon and Rogers’ systematic review further reported that within published literature on the general population, fear of dental injections ranges anywhere from 11.7%−91%, with Wright and colleagues identifying a prevalence of 71% [1,11]. It is therefore imperative that these children are identified and managed appropriately. The aim of this study is to identify whether dentally anxious children report fear of needles and explore how these children experience a fear of dental injections and other needles (such as intravenous cannulation) in a dental setting. Outside of professional psychological treatment, there are currently no interventions available for children with needle fear which have been developed with them, and are based on strategies with proven effectiveness such as cognitive behavioural therapy [12]. There is a paucity of literature exploring children’s needle fear, which hinders the development of such resources.

## 2. Materials and Methods

### 2.1. Secondary Analysis

**Figure 1:**
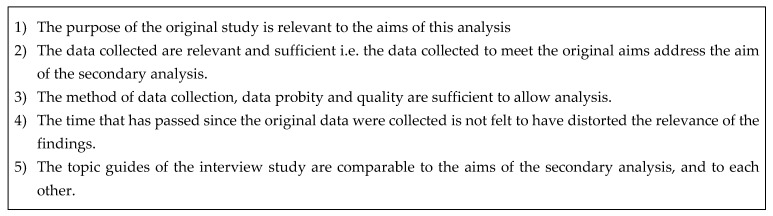
Inclusion criteria—assessment of comparability of qualitative data for secondary analysis (adapted from Stewart and Kamins, as used by Etkind et al., p. 173) [14].

Secondary analysis of qualitative data involves using pre-existing data. This can be to investigate new research questions, verify existing research and synthesise knowledge from previous studies [13]. In this study supplementary analysis of multiple data sets was used. Supplementary analysis is a type of secondary analysis used for ‘a more in-depth focus on an emergent issue or aspect of the data which was not addressed, or only partially addressed, by the primary research’ [13]. Data from different studies must be carefully assessed to establish whether it can be subject to supplementary analysis. Suitability of the data was assessed with reference to five criteria (Figure 1):

The researchers had access to interview data from two previous projects carried out in two sites which met these inclusion criteria. The first study sought to explore children’s experiences of dental anxiety using a cognitive behavioural therapy assessment model (hereafter referred to as the dental anxiety study [15]. The inclusion criteria for the study was children aged 11–16 who self-reported being dentally anxious, with no record of severity. The second study aimed to develop a decision aid for children faced with the decision to undergo dental treatment with inhalation sedation, intravenous sedation or general anaesthesia (hereafter referred to as the decision aid study) [16]_._ The inclusion criteria for this study was children aged 10–16 who had undergone dental treatment with sedation or general anaesthetic. Participants from both studies had given consent to use of their data for future research.

### 2.2. Sampling Selection

Two datasets containing thirteen (dental anxiety study) and eleven (decision aid study) transcripts were eligible for inclusion in the analysis. Transcripts were searched for references to ‘needle’ and ‘injection’. One transcript from the dental anxiety study was excluded as these topics were not discussed. One transcript from the decision aid study was not available at the time secondary analysis was carried out. The final sample of transcripts comprised twelve from the dental anxiety study and ten from the decision aid study.

### 2.3. Analysis 

Interview transcripts were analysed using a framework approach [17,18]. In this approach, recurring ideas in the data are identified and organised into a thematic framework. Analysis focused only on the sections of the transcripts which referred to needles or injections. This was to allow the identification of recurring themes. Two researchers established familiarisation with the data and worked together to identify significant ideas in the data relating to needles and injections (FN and JK). These were then organised into a theoretical framework of themes and sub-themes, which was used as a coding frame. Both researchers (FN and JK) applied the coding frame to ten per cent of the transcripts (n = 2). Following discussion, the researchers resolved minor discrepancies and made amendment to the coding frame. FN then applied the revised coding frame to the remaining transcripts. The coding frame was populated with examples from the original interviews to support the themes and sub-themes and discussed with the research team, who are familiar with the data from the original projects (ZM, HR & JH). Themes and sub-themes were refined through these discussions.

## 3. Results

### 3.1. Demographics

A total of 19 female (86%) and 3 male (14%) participants were included, with ages ranging from 10–15 years at the time of interview (see Table 1).

### 3.2. Analysis

The analysis revealed five main themes: feelings about needles; nature of fear; context of fear; consequences of fear; controlling the process. Within these main themes, further sub-themes are also discussed. The identified themes and sub-themes are shown below (Table 2). Themes and sub-themes are then outlined and illustrated with quotations from both studies. Participants are identified by age, gender and study, e.g., 14M, DenAnx (14 year old male in Dental Anxiety study); 15F, DecAid (15 year old female in Decision Aid study). 

#### 3.2.1. Feelings about Needles

This theme described the range of feelings expressed from negative to positive feelings, with some contradictory feelings reported. Children and adolescents expressed a dislike of needles or injections, and admitted that they were frightened of them. It was clear that for some participants the concept of having an injection or needle-based intervention was highly anxiety-provoking which in some cases may have been due to fear of the unknown:

‘I would rather be having my leg cut off than a little needle.’

(14M, DenAnx)

‘For me just because it’s the, it’s not the teeth coming out that I’ve got to get over it’s the injection bit.’

(15F, DecAid)

Despite the negative feelings, some children felt more positively about the prospect of a needle or showed ambivalence:

Interviewer: So are you alright with injections in your arm then?

‘Yeah, because I’ve had 5 already.’

(13F, DenAnx)

Children were more likely to express positive or ambivalent feelings towards needles if they had received multiple exposures previously:

‘Once I’ve done the first one, I can’t really say I won’t do the next.’

(14F, DenAnx)

#### 3.2.2. Nature of Fear

This theme explored the aspects of needles or injections which provoked fear in the participants with three sub-themes identified.

#### 3.2.3. Appearance of the Needle

The appearance of the needle was commonly identified as a source of anxiety, with children imagining what they might look like without having seen one before. Children reported anticipating that the needle used for dental injections would be large:

‘Like a normal injection but a bit bigger.’

(10F, DenAnx)

‘I had a picture like a big needle.’

(11F, DenAnx)

They also expressed a fear of seeing the sharp end of the needle, or of sharp objects in general.

‘I could see like all like pointy and sharp things that made me pretty scared.’

(14M, DenAnx)

‘It’s the point on the needle you seen first.’

(15F, DecAid)

#### 3.2.4. Feel of the Needle

The feeling of being injected, or how this was imagined was identified as influencing participants’ anxiety. Children reported having felt pain at previous exposures, or perceiving that it would be painful:

‘It stings really badly like 10,000 bees stinging you inside your mouth.’

(14M, DenAnx)

‘It is going to really really hurt.’

(10F, DenAnx)

Children also described the feelings they experienced after the procedure was completed. For some children this was a further negative experience, but some children enjoyed the feeling (of numbness):

‘I liked it. I could not feel that part of my face. But I liked it.’

(14F, DenAnx)

#### 3.2.5. Relevance of Area of the Body 

There was variation between participants in the relevance of the area of the body where the needle was administered. For some participants their feelings were unrelated to the area (anatomical site) of administration, and they experienced the same feelings towards all forms of needle or injection:

‘Interviewer: Is it just injections inside your mouth?

Participant: Any injections. Like at school or anything.’

(14F, DenAnx)

Others related their fear to the area of the injection. This included both particularly disliking injections in the mouth, and preferring injections in the mouth to injections in the arm:

‘Why can’t they put it in my arm, and why does it have to go in my mouth.’

(11F, DenAnx)

‘I would rather have one in my mouth than in my arms.’

(14M, DenAnx)

‘Participant: I am not having a needle stuck in my vein.’

Interviewer: Would you be alright having a needle in your mouth?

‘Participant: Yeah. I’ve had it before so I know what it is like.’

(15F, DenAnx)

#### 3.2.6. Context of Fear 

This theme situated experiences of needle phobia in a wider context, including patients’ own biographies and social influences from parents, siblings and friends. In some cases, participants were able to appreciate that previous incidents had contributed to their current fear of needles, and their fear could be facilitated by having further exposures:

‘The second time I was more panicky because I knew what I was expecting, and I didn’t want to have it done again.’

(14F, DenAnx)

Participants suggested that knowledge shared by family and friends could have the consequence of initiating or worsening their own fear:

‘They were like it’s the biggest needle you’ll ever see so.’

(14F, DenAnx)

‘They’d go like the needle really hurts and stuff.’

(10F, DecAid)

#### 3.2.7. Consequences of Fear

This theme explored the effect the fear of needles had on the children, in relation to altering their ‘normal’ state in some way. There were three sub-themes – physical impacts, behavioural impacts and emotional impacts. Physical response to fear included sensations such as feeling faint or hot:

‘I sort of nearly fainted.’

(14F, DecAid)

Children reported displaying uncharacteristic behaviour when confronted by their fear of needles, in order to avoid the intervention:

‘And like she once tried to like give me a needle but I didn’t want it. So I like moved her arm.’

(14F, DecAid)

‘They had to like bring people to hold me down and stuff.’

(14M, DenAnx)

Emotional responses included embarrassment, anger, upset and concerns that others were judging them negatively because of their fear:

‘But before I came to the appointment I was very distressed. Just sort of, you know, knowing that I was going to have to have an injection, it was going to have to happen. Erm beforehand it did make me very, I don’t know the word, just distressed.’

(15F, DecAid)

‘People just get sick of me because I’m taking ages because I don’t want it.’

(11F, DenAnx)

#### 3.2.8. Controlling the Process

Generally when children felt they had control over what was going to happen to this reduced their fear. In contrast, previous experiences where children had felt a loss of control were reported as having contributed to their fear. This theme had three subthemes namely ‘awareness and information’, ‘preparation’ and ‘trust’.

#### 3.2.9. Awareness and Information

Control related to what children’s preferred level of awareness, both before and during the procedure. While some participants stated that they did want to be aware about what was happening with the needle, some stated the opposite and reported that they did not want to know:

‘Now I know when the needle is going to be going in and stuff like that. And that is the thing.’

(15M, DecAid)

‘What was good also was that they didn’t tell me when they were going to do the injection. So in my first injection the first time I just felt stinging, And then I said are you doing the injection, and they were like err no. And then I was sort of calmer because I didn’t know they were doing it.’

(14F, DenAnx)

During the procedure, some participants specifically wanted to be distracted, in order to remove focus from being injected with a needle. Music was a popular choice for method of distraction in this cohort.

Children expressed a range of views on whether seeing the needle worsened or improved their dental anxiety. Some children felt they wanted to see the needle during the procedure, in order to feel prepared and ultimately, in control:

‘To see like what size it was and what was going on in my mouth.’

(10F, DenAnx)

Other children reported that seeing the needle would exacerbate their anxiety:

‘If they went round the side of my mouth that’s alright because you can see the full thing when they just go over my head.’

(15F, DecAid)

Again, they wanted to control what they saw and suggested this would be beneficial.

#### 3.2.10. Preparation

The sub-theme of preparation related to the child’s desire to mentally prepare for the procedure, following information about what to expect from a parent or professional. Some children felt strongly that they would like time to prepare themselves prior to the appointment:

‘I was expecting like just 1 and then I had 4 so.’

(14F, DecAid)

Interviewer: ‘Would you like the dentist to say we are going to do an injection?’

Participant: ‘Yeah’.

(11F, DenAnx)

Other treatments can also help with relaxation, allowing participants to feel more prepared:

‘I’ve been able to prepare myself, I’ve been able to have the gas and air.’

(15F, DecAid)

#### 3.2.11. Trust

The necessity of trust emerged as an important and related sub-theme and highlighted the relationships between both professional and parent with the child. It was very important to all the participants that they could trust their dentist, and any loss of trust had the consequence of making it difficult to form a positive relationship with other healthcare workers and adults in general:

‘When I had my needle I said when I put my hand up can you stop, he said yeah, and then when I put my hand up they didn’t, they went on.’

(11F, DenAnx)

‘After that I didn’t trust anybody.’

(11F, DenAnx)

‘We got this crap one, she was, she was like proper evil.’

(14F, DecAid)

Some children expressed a desire to have their parents with them for their interventions, either just being present in the room or being able to hold their hands or touch them:

‘I have to have someone in the room with me, but not holding my hand or anything.’

(15F, DenAnx)

Having a trusted adult present could help to make the situation feel safer. However, some families acknowledged that their presence at the appointments may have had a negative effect on their children’s behaviour

## 4. Discussion

The aim of this study was to explore how dentally anxious children experience fear of needles. Children commented on the way in which fear was experienced, in some cases identifying the aspects of injections that were problematic (such as the look or feel of the needle, or noting that the bodily location of the injection was significant). This also included noting different types of responses to fear: physical, behavioural and emotional, which are often inter-related. Needle fear was situated in a wider context, with parents, siblings, friends and healthcare professionals forming part of children’s accounts. 

It was particularly noticeable that children identified various factors that could improve or worsen their fear in specific situations. This included preferences about how much they wanted to know, both before and during procedures. For example, children commented on whether or not it would be helpful to see the needle or be distracted during an injection. Having a better understanding of children’s preferences regarding injections is important for ensuring a positive experience of treatment. Such preferences could be understood in terms of ‘control’; participants wanted to be able to control the flow of information they received. Children dislike feeling ‘helpless’, and increased feeling of vulnerability or loss of control negatively impacts on their experiences in dental settings [15,19,20,21].This theme is also identified separately in relation to children’s needle fear within this cohort, and suggests that further work may be required to increase children’s perceptions of control in needle based procedures.

Within this analysis, trust between the child and their parents or other adults was identified as a particularly significant theme, affecting the majority of the cohort. The importance of oxytocin (peptide hormone and neuropeptide) in influencing social development and maintenance of trust is well documented in the literature [22] and it is known that a loss of trust results in decreased oxytocin levels, and therefore can increase anxiety and stress in the individual [23,24]. There is limited literature on the importance and effect of trust between children and dentists on their anxieties. Further research into these relationships, and the effect of trust and/or mistrust is required in order to understand children’s experiences of encounters with health professionals. 

Previous research into the perceptions of children with needle anxiety has identified similar themes around children seeking control, fear of pain, the impact of their relationship with both their parents and healthcare professionals, the importance of trust, altered behaviour and emotions and feelings of shame and embarrassment [25,26,27,28,29]. However, there is a need for further focused research to understand the relationship between dental anxiety and needle phobia and map out the different ways this can manifest. The original study on children’s experiences of dental anxiety was used to develop a guided self-help CBT resource to reduce dental anxiety [15]. A similar project would facilitate the creation of a CBT - based resource to reduce needle fear.

There are several limitations to be considered in regards to secondary analysis of data [30,31]. Within this study, the main limitation is that the information collected, while adequate for the purposes of the analysis, was collected in order to answer a different research question. This results in the inability to achieve true richness of data, as where the researchers would have pursued a line of questioning or requested clarification from the participants this did not occur, as it was not the primary purpose of the study. One example of this limitation relates to the question of how relevant a child’s age is with respect to the initiation and progression of needle fear? A second is that it was not specifically recorded which of the participants had undergone needle procedures before, and which kind/s. Further enquiry would be needed, involving children of a much wider age range, with a variety of experiences, to gain a valid insight into any potential associations. Another limitation of secondary analysis is that the researcher completing the analysis did not conduct the original interviews, and therefore may not have access to resources such as field notes, which will contain information related to context and impressions of the interviewer. To mitigate this as far as possible three of the authors of this paper were researchers in the original studies, and were able to provide context in the absence of field notes.

Data saturation is used within most qualitative research studies, to determine when enough participants have been consulted, and enough data collected. Theoretical saturation can be said to be achieved when there are no new ‘codes’ or ideas emerging from the data, or when the data collected is sufficient to support the generated theory [32]. Within the confines of this secondary data analysis, full data saturation is not possible- as secondary researcher, there is no option to continue fieldwork until this point is reached. In view of the limitations of secondary analysis, a vigorous approach to analysis has been employed, allowing a framework to be developed which accurately represents the available data. This framework could be refined through further research. The data also has inherent differences between the two studies, although they are analysed in conjunction, which should be acknowledged. The decision aid study data focuses mainly on the reasons for choosing a method of treatment, and explores anxiety more generally. Questions around needles are asked explicitly, and there are several instances where the interviewer clarified the point a child or parent had made, and also drew distinctions between intra-oral vs. other kind of needle exposures. 

The dental anxiety study is very focused on the child’s experiences of anxiety and other than where the child is explicit in their statement, it is not always possible to differentiate between needle and dental phobia. For both studies, parents were invited to be present and participate, which will have had an influence on the data obtained. It is thought that the absence of parents enables the collection of richer data [33]. In this analysis, the presence of parents allowed for the expression of the child’s needle fear in several instances, and allowed for some exploration of the parents experience of both their own and their child’s needle fear. The researchers acknowledge that it may however have stunted the participation of the child themselves.

## 5. Conclusions

In conclusion, the themes around needle fear identified in this cohort are similar to previously identified themes in studies relating to both needle and dental fear in children, particularly the desire to have control of healthcare interventions, and to trust healthcare providers. This area has not yet been fully explored, and due to the limitations of a supplementary analysis, data saturation was not reached for this study. It is interesting to reflect that despite advances in care for dentally-anxious children, including adjuncts such as topical anaesthetic, sedative agents and psychoeducational approaches, that needle fear remains a very real issue for some children.

Further primary research into this subject area would be beneficial. It was noted within the analysis that needle fear is not always obviously identified as either concomitant or entirely separate to dental anxiety in children, and therefore potentially not adequately managed. As part of further research into this subject area, this concept should be explored in more depth. 

## Figures and Tables

**Table 1:** Participant demographics.

Pseudonym	Sex (M/F)	Age at Interview	Original Study
Chloe	F	11	Dental anxiety
Samantha	F	15	Dental anxiety
Danielle	F	11	Dental anxiety
Amelia	F	14	Dental anxiety
Joe	M	12	Dental anxiety
Lucy	F	13	Dental anxiety
Olivia	F	14	Dental anxiety
Sophie	F	12	Dental anxiety
Katy	F	10	Dental anxiety
Ella	F	14	Dental anxiety
Claire	F	14	Dental anxiety
Michael	M	14	Dental anxiety
WK	F	14	Decision aid
WJL	F	15	Decision aid
GL	F	15	Decision aid
MA	F	13	Decision aid
LJ	F	12	Decision aid
VL	F	14	Decision aid
WA	M	15	Decision aid
SC	F	13	Decision aid
LD	F	10	Decision aid
LK	F	15	Decision aid

**Table 2:** Themes and sub-themes.

Themes	Subthemes
Feelings about needles	
Nature of fear	Appearance of the needle
	Feel of the needle
	Relevance of area of the body
Context of fear	Origin of fear
	Social influences
Impacts of fear	Physical
	Behavioural
	Emotional
Controlling the process	Information and awareness
	Preparation
	Trust
